# Complete Genome Sequences of Streptococcus pyogenes Serotype M3, M28, and M89 Strains Isolated from Human Patients in Japan, 1994 to 2009

**DOI:** 10.1128/MRA.01047-20

**Published:** 2020-10-15

**Authors:** Kazunori Murase, Takashi Nozawa, Chihiro Aikawa, Miki Nagao, Tadayoshi Ikebe, Akemi Yoshida, Taisei Kikuchi, Ichiro Nakagawa

**Affiliations:** a Department of Microbiology, Graduate School of Medicine, Kyoto University, Kyoto, Japan; b Clinical Laboratory Medicine, Graduate School of Medicine, Kyoto University, Kyoto, Japan; c Department of Bacteriology I, National Institute of Infectious Diseases, Tokyo, Japan; d Laboratory of Genomics, Frontier Science Research Center, University of Miyazaki, Miyazaki, Japan; e Department of Infectious Diseases, Faculty of Medicine, University of Miyazaki, Miyazaki, Japan; University of Delaware

## Abstract

Streptococcus pyogenes (group A Streptococcus [GAS]) is a major human pathogen that occasionally causes severe and life-threatening invasive diseases. Here, we report the complete genome sequences of four GAS strains of three M types, which were isolated from patients with severe invasive disease in Japan.

## ANNOUNCEMENT

Streptococcus pyogenes (group A Streptococcus [GAS]) is a major human pathogen that causes streptococcal pharyngitis, skin and soft tissue infections, and life-threatening conditions such as streptococcal toxic shock syndrome (STSS) (1). A large number of virulence-related genes are encoded in GAS genomes and are involved in host-pathogen interaction, colonization, immune invasion, and long-term survival within hosts, causing diverse symptoms (2). GAS strains are generally classified on the basis of serologic differences in the M protein, an antiphagocytic cell surface molecule. Although more than 100 distinct M types have been identified so far, particular M types (M1, M3, M4, M6, M12, M28, and M89) that are associated with invasive GAS diseases have been frequently isolated (3, 4). In addition, recent epidemiological evidence has revealed the rapid emergence of the GAS genotype *emm*89 as a leading cause of disease in the world, particularly in the United Kingdom and Canada (5, 6). Here, we report the complete genome sequences of GAS serotype M3, M28, and M89 strains, which were isolated from patients with severe invasive disease in Japan.

Of the four GAS strains, two National Institute of Health (NIH [Japan]) strains (NIH34 and NIH35) and two Kyoto University (KUN) strains (KUN-0012590 and KUN-0014944) were isolated from the clinical specimens of patients with severe disease in Kochi (7) and Kyoto in Japan, respectively (Table 1). All clinical samples were cultured on 5% sheep blood agar plates to isolate the GAS strains (8). These strains were incubated for 10 h at 37°C in Todd-Hewitt broth supplemented with 2% yeast extract. The bacterial cells were lysed with lysozyme and mutanolysin; then, the genomic DNA was extracted by phenol-chloroform extraction and ethanol precipitation (9). The extracted DNA was short- and long-read sequenced on the MiSeq (Illumina) and MinION (Oxford Nanopore Technologies) platforms, respectively. An Illumina library was prepared using the Nextera DNA library prep kit, and paired-end reads were generated using the MiSeq reagent kit v3-600. A MinION library was prepared from unsheared genomic DNA using the rapid barcoding kit (SQK-RBK004) according to the manufacturer’s instructions and sequenced with an R9.4.1 flow cell (FLO-MIN106). The Illumina data were preprocessed using Trimmomatic v0.36 to remove adapter and low-quality sequences (10) and used in a hybrid assembly with the MinION long reads using the Unicycler pipeline v0.4.7b with default parameters (11). The Unicycler pipeline automatically identifies and trims overlaps for circular genomes and rotates the genome to begin with the *dnaA* gene. The complete genome sequence was then annotated using DFAST v1.2.6 with default parameters (12). The assembly statistics, general genome information, and relevant characteristics are summarized in Table 1. This genome information will help in understanding the GAS virulence mechanisms involved in diverse disease types and facilitate future epidemiologic investigations.

**TABLE 1:** Assembly statistics and general genome information for S. pyogenes strains

Statistic or characteristic	Data for S. pyogenes strain:			
	NIH34	NIH35	KUN-0012590	KUN-0014944
Strain information				
Origin	Blood	Blood	Sputum	Blood
Clinical symptom^*a*^	STSS, NF	STSS	Pneumonia	Bacteremia
Yr of isolation	1994	1995	2007	2009
M type^*b*^	M3	M28	M89	M89
Assembly and genome statistics^*c*^				
No. of MinION reads	488,389	417,386	540,643	298,000
No. of MiSeq reads	1,043,504	1,066,124	1,033,760	1,223,775
Total no. of MinION bases	682,781,742	807,804,694	684,964,454	611,947,167
Total no. of MiSeq bases	294,812,074	302,301,351	291,936,329	345,293,722
Avg coverage (×)	514.37	598.97	526.75	547.81
Chromosome description				
Genome size (bp)	1,900,555	1,853,362	1,854,597	1,747,400
G+C content (%)	38.6	38.3	38.5	38.6
No. of CDSs^*d*^	1,866	1,750	1,780	1,611
Avg protein length (bp)	291.5	300.3	296.7	307.8
Coding ratio (%)	85.8	85.1	85.4	85.1
No. of rRNAs (*rrn* operons)	18 (6)	18 (6)	18 (6)	18 (6)
No. of tRNAs/tmRNAs^*e*^	67	66	67	67
No. of CRISPRs	0	2	2	3
GenBank accession no.	AP023387	AP023388	AP023389	AP023390

a STSS, streptococcal toxic shock syndrome; NF, necrotizing fasciitis.

b M type, serological type of cell surface M protein.

c All values are based on the output from DFAST.

d CDSs, coding sequences.

e tmRNAs, transfer-messenger RNAs.

### Data availability.

The complete genome sequences of the four S. pyogenes strains in this study have been deposited in DDBJ/EMBL/GenBank under the accession numbers shown in Table 1. The raw Illumina and MinION read data can be found in the NCBI SRA under the BioProject accession number PRJDB10330.

## References

[1] Cole JN, Barnett TC, Nizet V, Walker MJ 2011 Molecular insight into invasive group A streptococcal disease. Nat Rev Microbiol 9:724–736. doi:10.1038/nrmicro2648. 21921933

[2] Nakagawa I, Kurokawa K, Yamashita A, Nakata M, Tomiyasu Y, Okahashi N, Kawabata S, Yamazaki K, Shiba T, Yasunaga T, Hayashi H, Hattori M, Hamada S 2003 Genome sequence of an M3 strain of Streptococcus pyogenes reveals a large-scale genomic rearrangement in invasive strains and new insights into phage evolution. Genome Res 13:1042–1055. doi:10.1101/gr.1096703. 12799345 PMC403657

[3] Gherardi G, Vitali LA, Creti R 2018 Prevalent emm types among invasive GAS in Europe and North America since year 2000. Front Public Health 6:59. doi:10.3389/fpubh.2018.00059. 29662874 PMC5890186

[4] Musser JM 2020 Molecular mechanisms contributing to fuzzy epidemics caused by group A *Streptococcus*, a flesh-eating human bacterial pathogen. Trans Am Clin Climatol Assoc 131:356–368. 32675873 PMC7358509

[5] Shea PR, Ewbank AL, Gonzalez-Lugo JH, Martagon-Rosado AJ, Martinez-Gutierrez JC, Rehman HA, Serrano-Gonzalez M, Fittipaldi N, Beres SB, Flores AR, Low DE, Willey BM, Musser JM 2011 Group A Streptococcus emm gene types in pharyngeal isolates, Ontario, Canada, 2002–2010. Emerg Infect Dis 17:2010–2017. doi:10.3201/eid1711.110159. 22099088 PMC3310556

[6] Turner CE, Abbott J, Lamagni T, Holden MTG, David S, Jones MD, Game L, Efstratiou A, Sriskandan S 2015 Emergence of a new highly successful acapsular group A Streptococcus clade of genotype emm89 in the United Kingdom. mBio 6:e00622-15. doi:10.1128/mBio.00622-15. 26173696 PMC4502227

[7] Inagaki Y, Konda T, Murayama S, Yamai S, Matsushima A, Gyobu Y, Tanaka D, Tamaru A, Katsukawa C, Katayama A, Tomita M, Fuchi Y, Hoashi K, Watanabe H 1997 Serotyping of Streptococcus pyogenes isolated from common and severe invasive infections in Japan, 1990–5: implication of the T3 serotype strain-expansion in TSLS. The Working Group for Group A Streptococci in Japan. Epidemiol Infect 119:41–48. doi:10.1017/s0950268897007644. 9287942 PMC2808821

[8] Spellerberg B, Brandt C 2016 Laboratory diagnosis of Streptococcus pyogenes (group A streptococci) *In* Ferretti JJ, Stevens DL, Fischetti VA (ed), Streptococcus pyogenes: basic biology to clinical manifestations, University of Oklahoma Health Sciences Center, Oklahoma City, OK. 26866238

[9] Borek AL, Obszańska K, Hryniewicz W, Sitkiewicz I 2012 Detection of Streptococcus pyogenes virulence factors by multiplex PCR. Virulence 3:529–533. doi:10.4161/viru.21540. 23076284 PMC3524156

[10] Bolger AM, Lohse M, Usadel B 2014 Trimmomatic: a flexible trimmer for Illumina sequence data. Bioinformatics 30:2114–2120. doi:10.1093/bioinformatics/btu170. 24695404 PMC4103590

[11] Wick RR, Judd LM, Gorrie CL, Holt KE 2017 Unicycler: resolving bacterial genome assemblies from short and long sequencing reads. PLoS Comput Biol 13:e1005595. doi:10.1371/journal.pcbi.1005595. 28594827 PMC5481147

[12] Tanizawa Y, Fujisawa T, Nakamura Y 2018 DFAST: a flexible prokaryotic genome annotation pipeline for faster genome publication. Bioinformatics 34:1037–1039. doi:10.1093/bioinformatics/btx713. 29106469 PMC5860143

